# Testing field adaptation strategies for delaying grape ripening and improving wine composition in a cv. Macabeo Mediterranean vineyard

**DOI:** 10.3389/fpls.2023.1155888

**Published:** 2023-04-25

**Authors:** Ignacio Buesa, Antonio Yeves, Diego Guerra, Felipe Sanz, Camilo Chirivella, Diego S. Intrigliolo

**Affiliations:** ^1^ Research Group on Plant Biology under Mediterranean Conditions, Department of Biology, University of the Balearic Islands, Palma, Spain; ^2^ Instituto Valenciano de Investigaciones Agrarias, Sustainable Agriculture Center, Unidad Asociada al Centro Superior de Investigaciones Científicas (CSIC) “Riego en la Agricultura Mediterránea”, Valencia, Spain; ^3^ Department of Ecology (CSIC, UV, GV), Centro de Investigaciones sobre Desertificación (CIDE), Valencia, Spain; ^4^ Instituto Tecnológico de Viticultura y Enología, Servicio de Producción Ecológica, Innovación y Tecnología, Valencia, Spain

**Keywords:** climate change, double-pruning, phenology, shading nets, vine performance, water stress

## Abstract

Under semiarid and warm climates, field practices for climate change adaptation have to be defined in order to modulate grape composition according to the desired wine styles. Under this context, the present study investigated several viticulture practices in cv. Macabeo for Cava production. The experiment was carried out over 3 years in a commercial vineyard located in the province of Valencia (eastern Spain). The techniques tested were (i) vine shading, (ii) double pruning (bud forcing), and (iii) the combined application of soil organic mulching and shading, all of them tested against a control. Double pruning significantly modified phenology and grape composition, improving the wine alcohol-to-acidity ratio and reducing the pH. Similar results were also achieved by shading. However, the shading strategy did not significantly affect yield, unlike double pruning, which reduced vine yield even in the year following its application. Shading alone or in combination with mulching significantly improved the vine water status, suggesting that these techniques can also be used to alleviate water stress. Particularly, we found that the effect of soil organic mulching and canopy shading on stem water potential was additive. Indeed, all the techniques tested were useful for improving wine composition for cava production, but double pruning is only recommended for premium Cava production.

## 1 Introduction

Climate change projections for the 21st century indicate that Mediterranean-like areas are especially vulnerable to the potential effects of temperature, shifts in rainfall patterns, and the frequency of extreme events (IPCC et al., 2014). Grapevine yield and quality depend on complex interactions between temperature, soil water availability, plant material, and field practices (van Leeuwen et al., 2019). For instance, a study conducted for 50 years across many European regions and cultivars showed that phenological timing has advanced, between 6 and 18 days depending on the cultivar (Jones et al., 2005; van Leeuwen et al., 2019). Also, a great spatial and temporal variability in temperature and phenology within a region has been reported (Neethling et al., 2019; de Rességuier et al., 2020). Overall, the combined effect of advanced phenology and increased temperatures and reference evapotranspiration (ET_o_) has resulted in warmer and drier conditions during the grape-ripening phase (Previtali et al., 2022). On the one hand, under warmer conditions, grapes have an increased sugar concentration, which results in a higher alcohol content in wines, and decreased organic acid content, while aromas and aroma precursors are dramatically changed (Duchêne and Schneider, 2005; Neethling et al., 2012; van Leeuwen and Destrac-Irvine, 2017). Moreover, increased soil water deficit and higher ET_o_ are expected to reduce vine vigor and yield. In this context, adaptation strategies that minimize these effects on vine performance and on berry composition and, consequently, on wine quality must be achieved.

Possible adaptation strategies to climate change could include earlier harvesting, although this is not feasible as grapes would not have the correct phenolic maturity (Sadras and Moran, 2012), the relocation of vineyards to cooler locations, in altitude or latitude (Jones et al., 2005; Ollat et al., 2016), and changing the current genetic material used (grapevine varieties, clones, and rootstocks) (Schultz, 2000; Medrano et al., 2015; van Leeuwen and Destrac-Irvine, 2017). In addition, other strategies include changes in field management techniques such as grapevine architecture, light interception modulation, the adjustment of the source-to-sink balance, canopy management, soil management, irrigation, and shifting vine phenology (Intrigliolo and Castel, 2011; Ollat et al., 2016; Martínez-Moreno et al., 2019; Buesa et al., 2020a; Buesa et al., 2020b; Buesa et al., 2021c; Naulleau et al., 2021; Previtali et al., 2022). Regardless of the strategy chosen, the success of the adaptation techniques to climate change strongly depends on the interaction of ecological and socioeconomic factors assessed locally (Lereboullet et al., 2013).

The present work aimed at evaluating shading, double pruning, and shading + mulching as adaptation strategies in a ‘Macabeo’ vineyard. This cultivar can be used to make still and sparkling wines. The latter, if prepared with traditional methods, can be called Cava. The Macabeo cultivar (syn. ‘Viura’) ripens early as compared to other white cultivars authorized by the Cava designation of origin. It is usually harvested early in order to maintain a good balance between alcohol and acidity as low levels of acidity would lead to a lack of brightness and aromas (Sweetman et al., 2009). Moreover, total acidity (TA) is directly related to the microbiological stability of the wine and, therefore, to the aging capacity of sparkling wines, carbonic maceration, or malolactic fermentation (Poni et al., 2018). However, under the projected climate change scenario, field strategies must be adopted for this cultivar to be used for premium sparkling wine production (Jones et al., 2014). Knowledge about the relationship between microclimatic conditions (mainly temperature and light environment around the clusters) and grape composition is scarce.

The first adaptation strategy evaluated was the modulation of light intercepted through the use of shading nets due to their effects on canopy microclimate and thus in determining grape composition (Greer et al., 2010; Caravia et al., 2016). The reduction in the amount of solar radiation intercepted by the vineyard can reduce grapevine photosynthetic capacity and canopy transpiration, slowing down the ripening process and alleviating vine water stress (Zufferey et al., 2000; Palliotti et al., 2014; Basile et al., 2015; Martínez-Lüscher et al., 2020). Thus, both water and heat stress can be alleviated by regulating the sunlight intercepted by vineyards (Intrieri et al., 1998; Williams and Ayars, 2005). Moreover, the reductions in light interception during grape ripening greatly affect leaf and cluster microclimate conditions, affecting vine physiology and grape ripening (Medrano et al., 2012; Manja and Aoun, 2019). This could mitigate the excessive exposure to sunlight of the cluster and overheating, reducing subsequent grape acidity catabolism and aromatic composition alterations, as well as berry sunburn and shriveling (Keller, 2010; Caravia et al., 2016; Pons et al., 2017; Gambetta et al., 2021). The exposure of the cluster to high solar radiation could increase the polyphenol content in the berry, which is considered a drawback for white wine production as it affects the final color of the wine (van Leeuwen and Destrac-Irvine, 2017).

The second adaptation strategy evaluated was a double pruning technique (i.e. bud forcing) to delay vine phenology for several months and thus grape ripening timing as well (Gu et al., 2012; Martínez-Moreno et al., 2019; Gutiérrez-Gamboa et al., 2021). It consists of green cane pruning and removing all the non-perennial parts (lateral branches, leaves, and clusters), to force the growth of the primary buds formed for the subsequent season. This technique can greatly reduce or not affect yield at all, as compared to unforced vines, depending on the timing and number of parts retained. However, interestingly, grapes from double-pruned vines show higher acidities, a lower pH, and much higher phenolic content as compared to the berries from the unforced vines (Cabral et al., 2023). Overall, a further postponement of the grape-ripening process seems to be a quite promising tool for addressing the detrimental effects of high temperatures and ET_o_ on fruit and wine quality in warm and semiarid regions. Nevertheless, large aroma differences in wine typicity are expected under such changes in the weather conditions during ripening (Jones et al., 2014; Vilanova et al., 2019; Buesa et al., 2021a).

Furthermore, the combination of adaptation strategies may have additive effects, which might provide better solutions for adapting to climate change (Naulleau et al., 2021). In this sense, the combination of shading nets and organic mulch application to the soil may improve the vine water status due to the reduction in evaporation from soil mulching (Buesa et al., 2021b). Even under drip irrigation, soil evaporation can be, in fact, an important component of the vineyard water balance, accounting for up to 30% of the entire vineyard evapotranspiration when canopies were managed with vertical shoot positioning (López-Urrea et al., 2020).

The study hypothesis is that the adaptation strategies proposed can improve grape and wine composition while preserving yield, through both the amelioration of vineyard microclimate, and the improvement of the vine water status.

## 2 Materials and methods

### 2.1 Vineyard site

The trial was carried out from 2017 to 2019 in a commercial ‘Macabeo’ vineyard (*Vitis vinifera* L.) located in Requena, Valencia, Spain (39°30´02´´N, 1°13´48´´W; elevation 740 m a.s.l.). The vineyard was planted in 2002 with 161-49C rootstock at a spacing of 2.5 × 1.5 m (2,666 vines ha^-1^). This rootstock (*V. riparia* × *V. berlandieri*) is well adapted to calcareous clay soils; it confers medium vigor to the scion, provides a steady yield, and promotes early ripening (Chomé Fuster et al., 2003). Vines were winter-pruned to a 16-bud count per vine on a bilateral cordon de Royat and trained to a vertical trellis system oriented in the south–west north–east direction.

The farm’s soil was a Typic Calciorthid according to the Soil Taxonomy (Soil Survey Staff, 1999), with a clay loam-to-light clay texture according to the U.S. Department of Agriculture (USDA), highly calcareous (37%) and with low fertility (<1% in organic matter). Soil depth was greater than 2 m with 200 mm m^-1^ of available water capacity. The climate in this area is classified as semiarid hot-summer Mediterranean (Rodríguez-Ballesteros, 2016); the heliothermal index of Huglin (Huglin, 1978) was 2,291°C, corresponding to a temperate-warm viticultural climate, with cool nights and moderately dry according to the classification system for grape-growing regions proposed by Tonietto and Carbonneau (2004). At the experimental site, the annual average values (for the 2002–2016 period) of the reference evapotranspiration (ET_o_) and rainfall were 1,114 and 402 mm, respectively. Sustained deficit irrigation was applied from early June to the end of September by the vineyard manager according to standard practices carried out in the area of study by the irrigation association, which normally delivers 60–100 mm per season of irrigation water (Ramírez-Cuesta et al., 2023). Drip irrigation was applied using a single pipe per vine row with two 4 L h^-1^ compensated emitters per plant.

### 2.2 Experimental design

The experiment consisted of three treatments: (i) control, winter-pruned; (ii) shading, the application of shading nets 1 m over the canopy to reduce photosynthetic active radiation on the vines by 50% once the phenological stage of pea size was reached (**Supplemental Figure 1A**); and (iii) double pruning and winter pruning plus severe green pruning 20 days after bloom (**Supplemental Figure 1B**). In 2019, in the double pruning treatments, the vines were pruned as the control in order to assess the carry-over effect of the application of the technique in the previous seasons on vine performance.

The experimental design was a complete block layout with four replicates. Each block comprised 10 rows of 7 vines. The shading treatment was located in the middle of each replicate due to the installation of the metal structure. Each experimental unit (EU), a combination of block × treatment, consisted of 10 experimental vines plus the surrounding perimeter vines acting as borders.

Moreover, in 2018 and 2019, the combination of field adaptation strategies was assessed only on the effects on the water status of the grapevine. This was done by including a subtreatment consisting of an organic mulch covering the soil in the drip zone under shaded conditions (shading + mulching). This subexperiment was carried out on four replicates of four vines within the borders of the shading treatment. Mulching consisted of the application of mechanically chopped vine prunings in the vine rows. This organic mulch was 1 m wide on the soil and 3–5 cm thick.

### 2.3 Field measurements and laboratory determinations

Weather data were recorded at an automated meteorological station located within the farm perimeter. Reference evapotranspiration (ET_o_) was calculated with the Penman–Monteith equation (Allen et al., 1998). Time periods were measured as days of the year (DOYs). Midday Ψ_stem_ was determined throughout the season with a pressure chamber (Model 600, PMS Instrument Company, USA) on bag-covered leaves from two representative vines per EU at noon (measurements were carried out between 11:30 and 12:30 solar time). The leaves used for these measurements were located on the west side of the row and enclosed in hermetic plastic bags covered with aluminum foil for at least 1 h prior to the measurements (Choné et al., 2001).

In addition, the additive effect of water deficit duration and intensity was accounted for by the water stress integral (S_Ψ_) computed as the sum of Ψ_stem_ measured every day during a given period (Myers, 1988). It was calculated from the Ψ_stem_ values over the veraison to harvest periods, subtracting those with the least negative value registered in a fully irrigated vineyard by Buesa et al. (2017) (−0.24 MPa) and considering the number of days in between measurements.

Harvest was carried out in each treatment aiming a level of 18°–19° Brix in the must, which is the standard for the traditional method of making sparkling wines (Jones et al., 2014). Therefore, harvest was performed at different dates within the year depending on the treatment. Grape yield, the number of clusters per vine, average cluster mass, and shoot fruitfulness (the number of clusters per shoot) were determined at harvest on each experimental vine. Shoots per vine were counted at the end of the season, and pruning fresh mass was weighted on each experimental vine. In addition, the yield-to-pruning ratio, known as the Ravaz index, was calculated to estimate the vine balance.

### 2.4 Grape and wine composition

Berry ripening evolution was assessed approximately every 10 days, starting from shortly before veraison to harvest. Berry fresh mass was determined on two random samples of 100 berries per EU. Berry samples were crushed and hand-pressed through a metal screen filter and used to evaluate technologically defined maturity. Must total soluble solids (TSSs) were determined by refractometry (PR-101, Series Palette, Atago Co, LTD, Japan); pH and titratable acidity (TA) were measured with an automatic titrator (Metrohm, Herisau, Switzerland). Must was titrated with a 0.1 N solution of NaOH to an end point of pH 7, and results were expressed in tartaric acid equivalents. Malic acid and tartaric acid were determined by colorimetric methods using an automated sequential analyzer (Easychem Plus, Systea, Anagni, Italy). All analytical determinations in musts were performed in duplicate. In addition, the TSS-to-TA and tartaric-to-malic acid ratios were calculated.

Wines were separately made from the grapes of each EU at the experimental winery. Thus, in the 2017 season, 12 vinifications were performed. However, in 2018 and 2019, the double pruning treatment was not vinified as in 2018. The grapes from this treatment did not reach commercial TSS content, while in 2019, only the carry-over effect of this technique on vine performance was evaluated. Grapes were mechanically crushed, destemmed, pressed, and fermented at a temperature of approximately 22 °C in 20 L stainless-steel containers. In all the musts, K_2_S_2_O_5_ was added at a ratio of 10 g/100 L of must. Afterward, these were then inoculated with 20 g of commercial *Saccharomyces cerevisiae* yeast that was previously hydrated at 37°C for 30 min (FR Excellence, Lamothe-Abiet). All the wines were stored at approximately 20°C for 3–4 months before analytical determinations. Fourier-transform infrared spectroscopy (BACHUS II, TDI, Barcelona, Spain) was used for determining alcohol content, TA, pH, citric and lactic acids, and glycerol content. All analytical determinations in wine were done in duplicate.

### 2.5 Statistical analysis

A two-way analysis of variance (ANOVA) was used to test the effect of the treatment and year and the treatment per year interaction (T*year) on vine performance variables. As significant interactions between T*year were observed in most variables, one-way ANOVAs were used to assess the effect of the treatment within each year. Similarly, the effect of the treatment on grape and wine composition variables were assessed by a one-way ANOVA. In the case that the ANOVA detected significant effects (P < 0.05), a mean separation was performed with the Duncan multiple range test. The ANOVAs and *post hoc* tests were carried out using the Statgraphics Centurion XVI package (version 16.0.07) (Statgraphics Technologies, The Plains, VA, USA). Additionally, regressions were calculated using SigmaPlot (version 11.0) (Systat Software, San Jose, CA, USA).

## 3 Results

### 3.1 Climate and water relations

**Figure 1:**
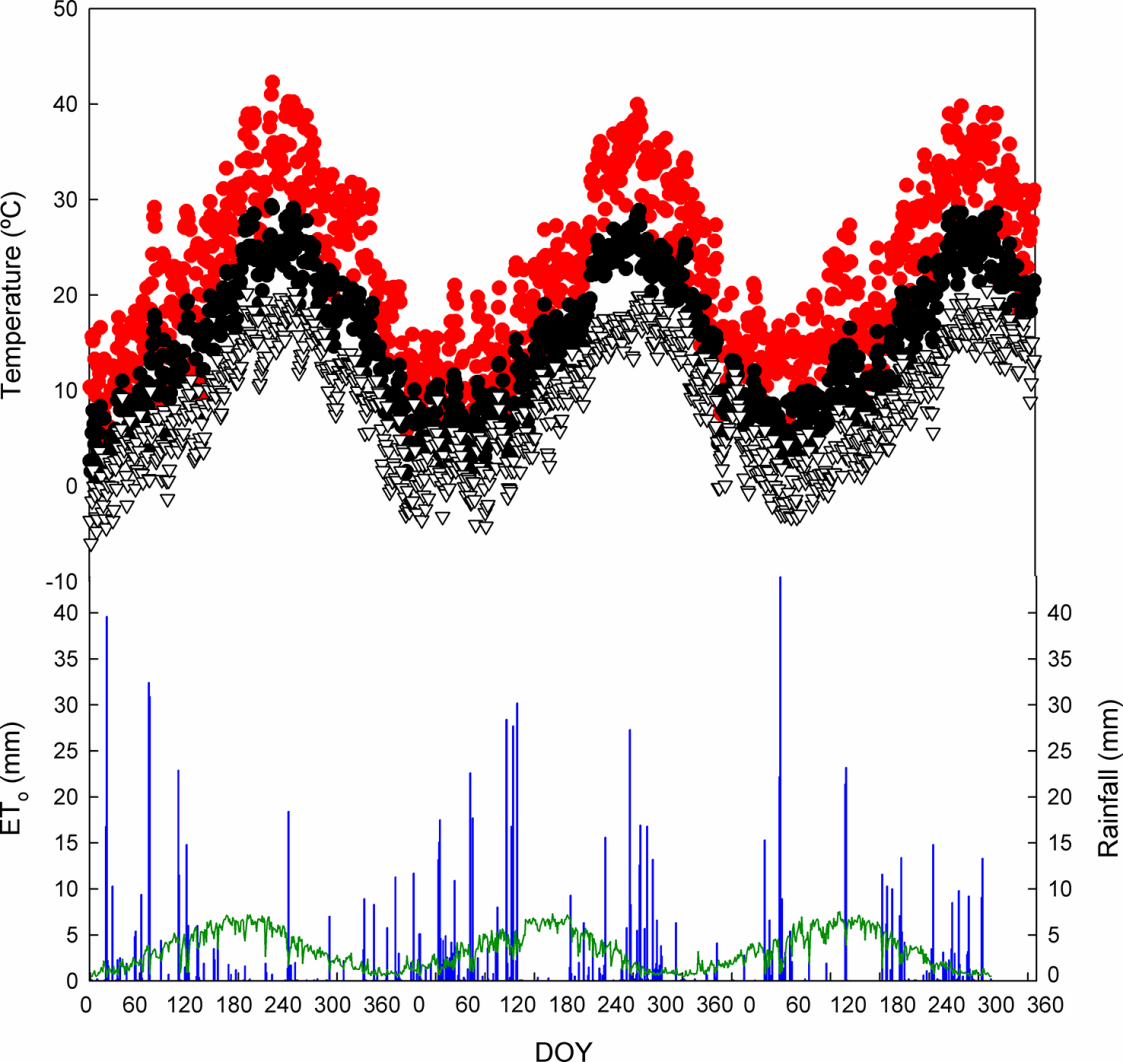
Seasonal patterns of the daily maximum air temperature (•), mean temperature (•), and minimum temperature (▽) in Requena, Valencia, Spain. Rainfall is represented with blue bars and reference evapotranspiration (ET_o_) with a green line. DOY, day of the year.

The results presented correspond to two dry years, 2017 and 2019, and a very wet one, 2018 (**Figure 1**). The values of rainfall were 322, 515, and 354 mm for 2017, 2018, and 2019, respectively, while the ET_o_ was 1,251, 1,144, and 1,218 mm, respectively. Maximum, minimum, and average temperatures showed a fairly similar pattern during the growing season (from April to October). On the other hand, from April to October, rainfall was 126, 314, and 250 mm in 2017, 2018, and 2019, respectively.

**Figure 2:**
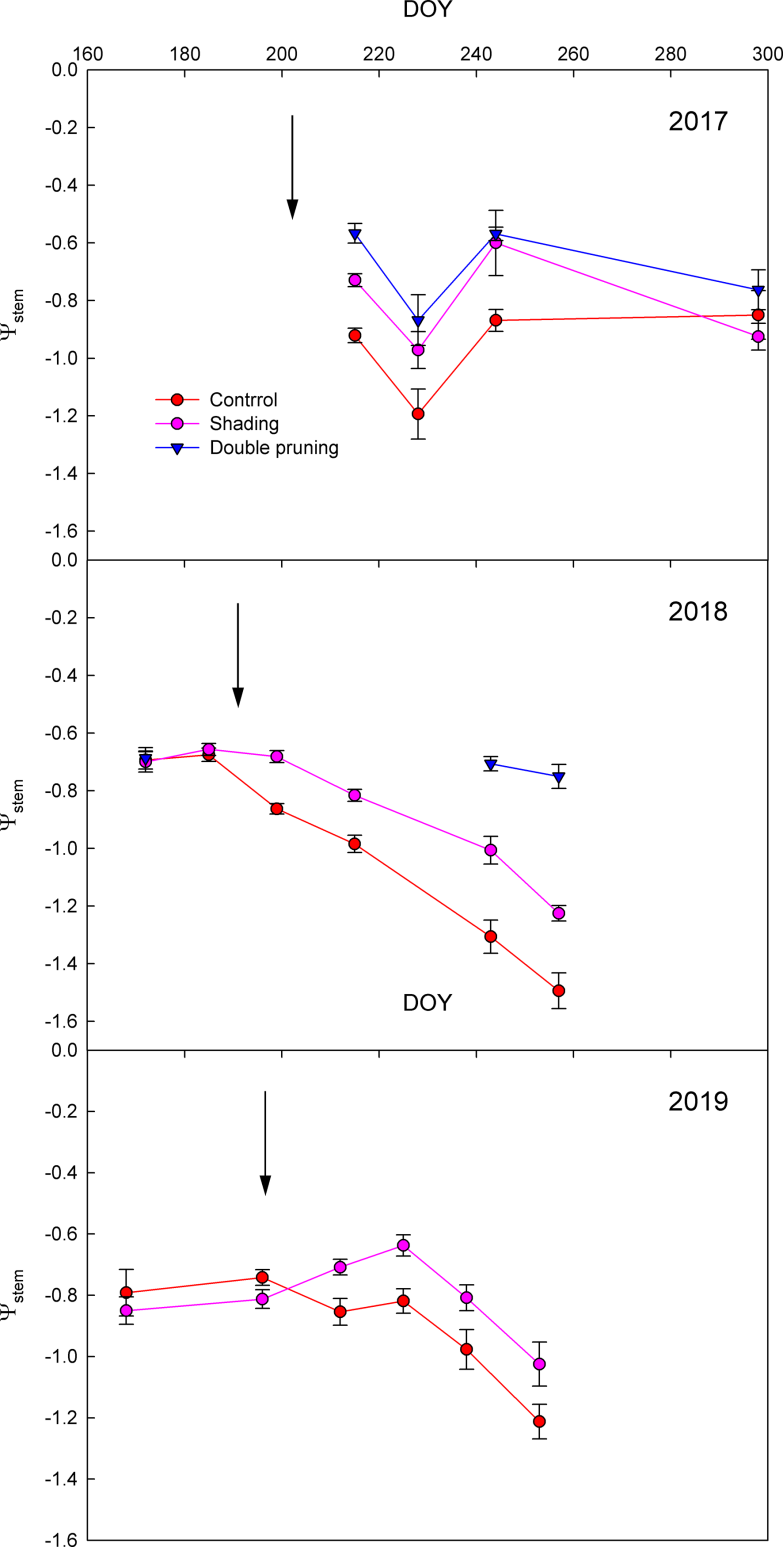
Effect of shading (•), double pruning (▼), and control (•) treatments on the seasonal evolution of the midday stem water potential (Ψ_stem_) of Macabeo grapevines in **(a)** 2017, **(b)** 2018, and **(c)** 2019 seasons. Data are the averages and standard errors of 16 leaves per treatment and date. The installation of nets (↓) is indicated.

The seasonal evolution of Ψ_stem_ showed that the vine water status was clearly affected by the different adaptation strategies (**Figure 2**). Both shading and double pruning treatments significantly reduced vine water stress as compared to that of the control in most of the measurement dates. The fast effect of the installation of the shading net on the Ψ_stem_ as compared to the control was remarkable (see arrows in **Figure 2**). The average Ψ_stem_ reduction in the shading treatments as compared to that of the control was 0.15, 0.23, and 0.17 MPa for 2017, 2018, and 2019, respectively. The reductions in Ψ_stem_ of the double pruning treatment as compared to the control were even greater (**Figure 2**).

**Figure 3:**
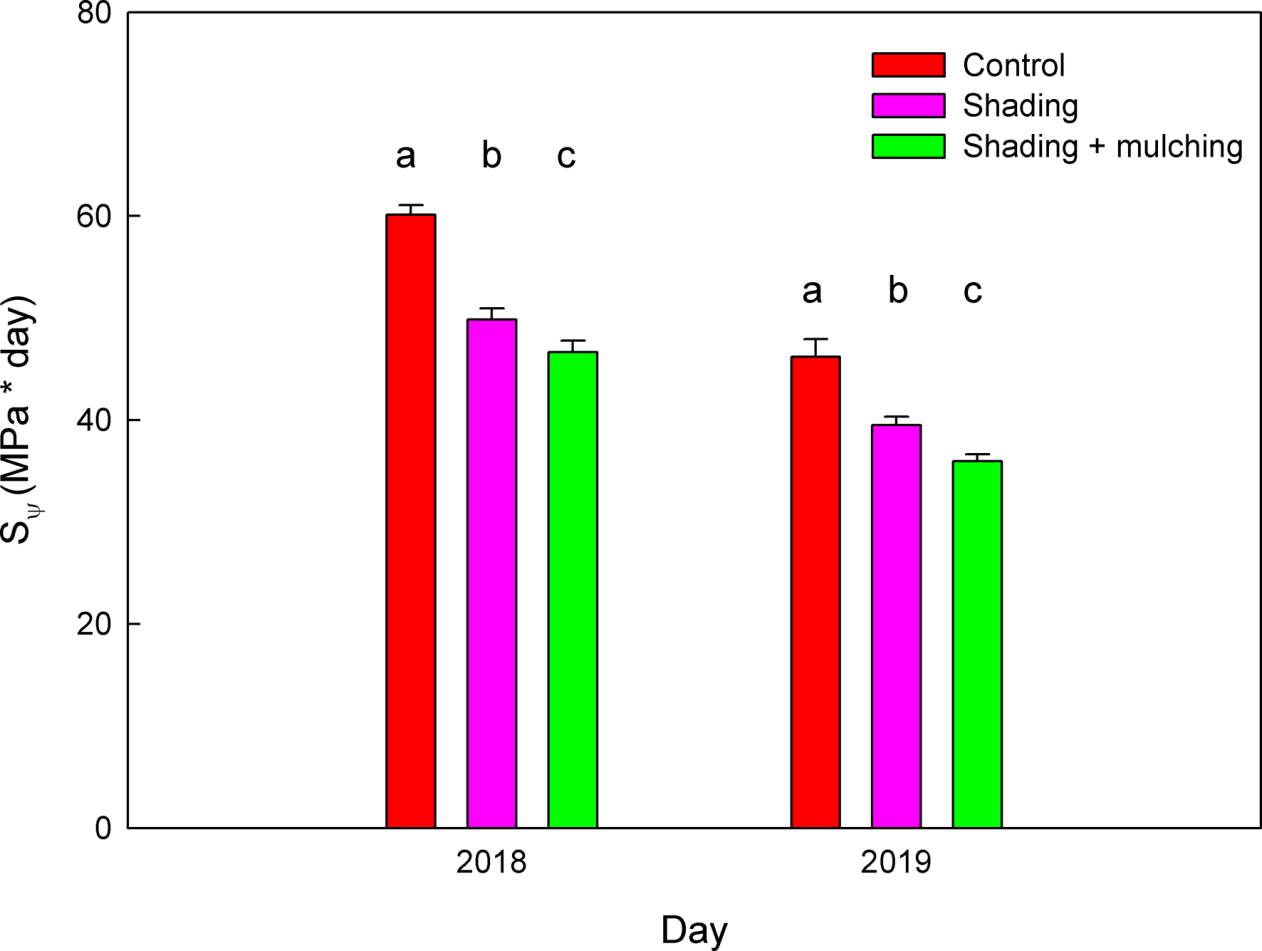
Values of the water stress integral (S_Ψ_) during the period from veraison to harvest during the 2018 and 2019 seasons for Macabeo grapevines subjected to different management strategies. Data are the average and standard errors of eight measurements per date and season (n = 24). Different letters mean a significant difference among treatments within each season (Duncan test; *p* < 0.05).

Moreover, the addition of mulching in the shading treatment significantly decreased the values of the S_Ψ_ during veraison to harvest in the 2018 and 2019 seasons as compared to the shading application alone (**Figure 3**). The reduction in S_Ψ_ of the shading + mulching treatment as compared to the control was 22%, while the one between the shading treatment and the control was only 16%.

### 3.2 Agronomic and physiological response

Differences in the number of shoots per vine among treatments were not consistent across seasons, with a significant T*year interaction observed (**Table 1**). The shading treatment, applied shortly before veraison, did not cause phenological differences with respect to the control. The double pruning treatment was effective in provoking the regrowth of the vine as no differences in the number of shoots per vine as compared to the control were found in any season. The double pruning treatment indeed showed a proper development of the entire growing cycle, although it delayed the phenology of the crop by several months.

**Table 1:** Average values of vegetative growth and yield components over the 3 years of the experiment in ‘Macabeo’ grapevines subjected to different treatments.

Parameter	Year	Treatments												Significance of effects		
		Control				Shading				Double pruning				Treat	Year	T*year
Shoots vine^-1^	2017	16.1	±	0.4	ab	15.7	±	0.4	a	17.0	±	0.4	b	0.152	<0.0001	<0.0001
	2018	16.9	±	0.4	b	15.6	±	0.4	a	16.8	±	0.4	b			
	2019	18.4	±	0.7	a	22.2	±	0.7	b	19.5	±	0.6	a			
Pruning mass (Kg vine^-1^)	2017	0.80	±	0.06	b	0.89	±	0.06	b	0.28	±	0.06	a	<0.0001	0.008	<0.0001
	2018	0.96	±	0.06	b	1.10	±	0.06	b	0.32	±	0.06	a			
	2019	0.87	±	0.05	b	0.85	±	0.06	b	0.68	±	0.06	a			
Clusters vine^-1^	2017	14.6	±	0.7	b	14.5	±	0.6	b	10.7	±	0.7	a	<0.0001	<0.0001	0.0001
	2018	21.3	±	0.8	c	18.9	±	0.7	b	11.6	±	0.7	a			
	2019	20.1	±	1.0	b	22.2	±	0.9	b	13.1	±	0.8	a			
Shoot fruitfulness (clusters shoot^-1^)	2017	0.91	±	0.04	b	0.91	±	0.04	b	0.62	±	0.04	a	<0.0001	<0.0001	0.061
	2018	1.06	±	0.05	b	1.16	±	0.04	b	0.81	±	0.04	a			
	2019	1.10	±	0.05	b	1.00	±	0.04	b	0.63	±	0.04	a			
Cluster mass (g)	2017	426	±	16	b	445	±	15	b	91	±	15	a	<0.0001	0.0005	<0.0001
	2018	438	±	14	c	336	±	13	b	70	±	13	a			
	2019	375	±	19	b	304	±	16	a	299	±	18	a			
Berry mass	2017	2.3	±	0.1	b	2.3	±	0.1	b	1.6	±	0.1	a	–	–	–
	2018	1.7	±	0.0	b	1.8	±	0.0	b	1.2	±	0.0	a			
	2019	1.9	±	0.1	a	1.8	±	0.1	a	–	–	–	–			
Yield (kg vine^-1^)	2017	6.2	±	0.3	b	6.4	±	0.3	b	1.0	±	0.3	a	<0.0001	<0.0001	<0.0001
	2018	9.3	±	0.3	c	6.3	±	0.3	b	0.8	±	0.3	a			
	2019	7.5	±	0.4	b	6.6	±	0.4	b	3.9	±	0.3	a			
Ravaz index	2017	8.6	±	0.7	b	8.2	±	0.7	b	4.2	±	0.7	a	<0.0001	0.07	0.0001
	2018	10.1	±	0.5	c	6.3	±	0.5	b	2.7	±	0.5	a			
	2019	9.0	±	0.5	b	7.9	±	0.5	ab	6.5	±	0.5	a			

Data are average and standard errors of four replicates per treatment and season (n = 4). Different letters mean a significant difference among treatments within each season (Duncan test; p < 0.05).

Vegetative vigor, measured through the pruning mass per vine, showed significant differences between treatments (**Table 1**). Control and shading treatments did not differ between them in this parameter. However, the pruning mass was significantly lower in the double pruning than in the other two treatments. Differences with the control were of 65% and 71% in 2017 and 2018, respectively. In 2019, when no second pruning was performed, the differences in pruning mass were only 22%, confirming the carry-over effects of the previous seasons of double pruning.

Regarding grape yield, differences between treatments were even higher than in vigor (**Table 1**). Significant differences between shading and control were found only in 2018, where the adaptation strategy reduced yield by 27%. The reductions in the double pruning treatment compared to both the control and shading were, in the two first experimental seasons, of 84% and 87%, respectively. The carry-over effect reduced by 47% in 2019. Differences in yield were mainly due to a reduction in the number of clusters per vine, as well as in cluster mass. Shading significantly reduced this parameter as compared to control, but only in 2018 (**Table 1**). Nevertheless, the ratio between the number of clusters and the number of shoots, known as shoot fruitfulness, did not significantly change in response to shading as compared to control, with it being 1.05 and 1.09 on average, respectively. The cluster mass in the shading treatment was similar to that of the control in 2017 and 2019 but significantly reduced in 2018. In this season, there was a general Botrytis affection in the clusters, and the shading treatment was more affected than the control. Regarding berry mass, there were no differences between shading and control treatments.

The double pruning technique reduced the number of clusters per vine in 2017 and 2018 as compared to that of the control. Thus, the fruitfulness in the double pruning treatment was significantly reduced by 62% on average, as compared to that of the control. Cluster mass was also significantly lower in the double pruning treatment than in the control, with reductions of 79% in the first two seasons and of 19% in 2019 (**Table 1**). Berry mass was, on average, 30% lower in the double pruning treatment as compared to the control (**Table 1**).

The Ravaz index was not affected by shading as compared to control, in 2017 and 2019, but it was significantly lower in 2018 due to the reduction in yield, whereas this index was significantly reduced by the double pruning in every season as compared to both control and shading treatments (**Table 1**).

### 3.3 Grape and wine composition

The control and shading treatments were harvested at the end of August or September, depending on the season (**Table 2**). The shading treatment was harvested between 4 and 8 days after the control, whereas the double pruning treatment was picked at the end of October or even in November. This means that harvest was delayed 43 and 62 days more than the shading treatment in 2017 and 2018, respectively. This harvest delay was related to the delays observed in grape veraison, with the control and shading veraison occurring in late July and early September, while the double pruning occurred during the first week of October.

**Table 2:** Harvest date and average values of berry composition at harvest over the 3 years of the experiment in Macabeo grapevines subjected to different treatments.

Parameter	Year	Treatments											
		Control				Shading				Double pruning			
Harvest date (DOY)	2017	233				237				299			
	2018	261				269				312			
	2019	252				252				–			
TSS (°Brix)	2017	19.6	±	0.4	a	18.4	±	0.4	a	21.3	±	0.4	b
	2018	18.4	±	0.3	b	15.6	±	0.3	a	14.9	±	0.3	a
	2019	17.9	±	0.4	a	16.7	±	0.4	a	–	–	–	–
pH	2017	3.28	±	0.02	b	3.27	±	0.02	b	3.15	±	0.02	a
	2018	3.18	±	0.02	b	3.29	±	0.02	c	3.12	±	0.02	a
	2019	3.39	±	0.02	b	3.25	±	0.02	a	–	–	–	–
TA (g/L)	2017	5.8	±	0.2	a	6.2	±	0.2	a	9.9	±	0.2	b
	2018	5.8	±	0.3	a	7.8	±	0.3	b	7.2	±	0.3	b
	2019	5.9	±	0.2	a	7.4	±	0.2	b	–	–	–	–
Tartaric acid (g/L)	2017	5.8	±	0.1	a	5.8	±	0.1	a	7.2	±	0.1	b
	2018	7.6	±	0.1	a	9.1	±	0.1	c	8.6	±	0.1	b
	2019	6.1	±	0.2	a	6.4	±	0.2	a	–	–	–	–
Malic acid (g/L)	2017	2.7	±	0.2	a	2.9	±	0.2	a	5.9	±	0.2	b
	2018	1.4	±	0.2	a	3.3	±	0.2	b	7.5	±	0.2	c
	2019	1.7	±	0.1	a	3.2	±	0.1	b	–	–	–	–
TSS-to-TA	2017	3.4	±	0.1	c	3.0	±	0.1	b	2.2	±	0.1	a
	2018	3.2	±	0.1	b	2.0	±	0.1	a	2.1	±	0.1	a
	2019	3.1	±	0.1	b	2.3	±	0.1	a	–	–	–	–
Tartaric/malic ratio	2017	3.9	±	0.3	c	2.3	±	0.3	b	1.2	±	0.4	a
	2018	5.8	±	0.4	c	2.8	±	0.4	b	1.1	±	0.4	a
	2019	3.7	±	0.2	b	2.0	±	0.2	a	–	–	–	–

Data are average and standard errors of four replicates per treatment and season (n = 4). Different letters mean a significant difference among treatments within each season (Duncan test; p < 0.05).

**Figure 4:**
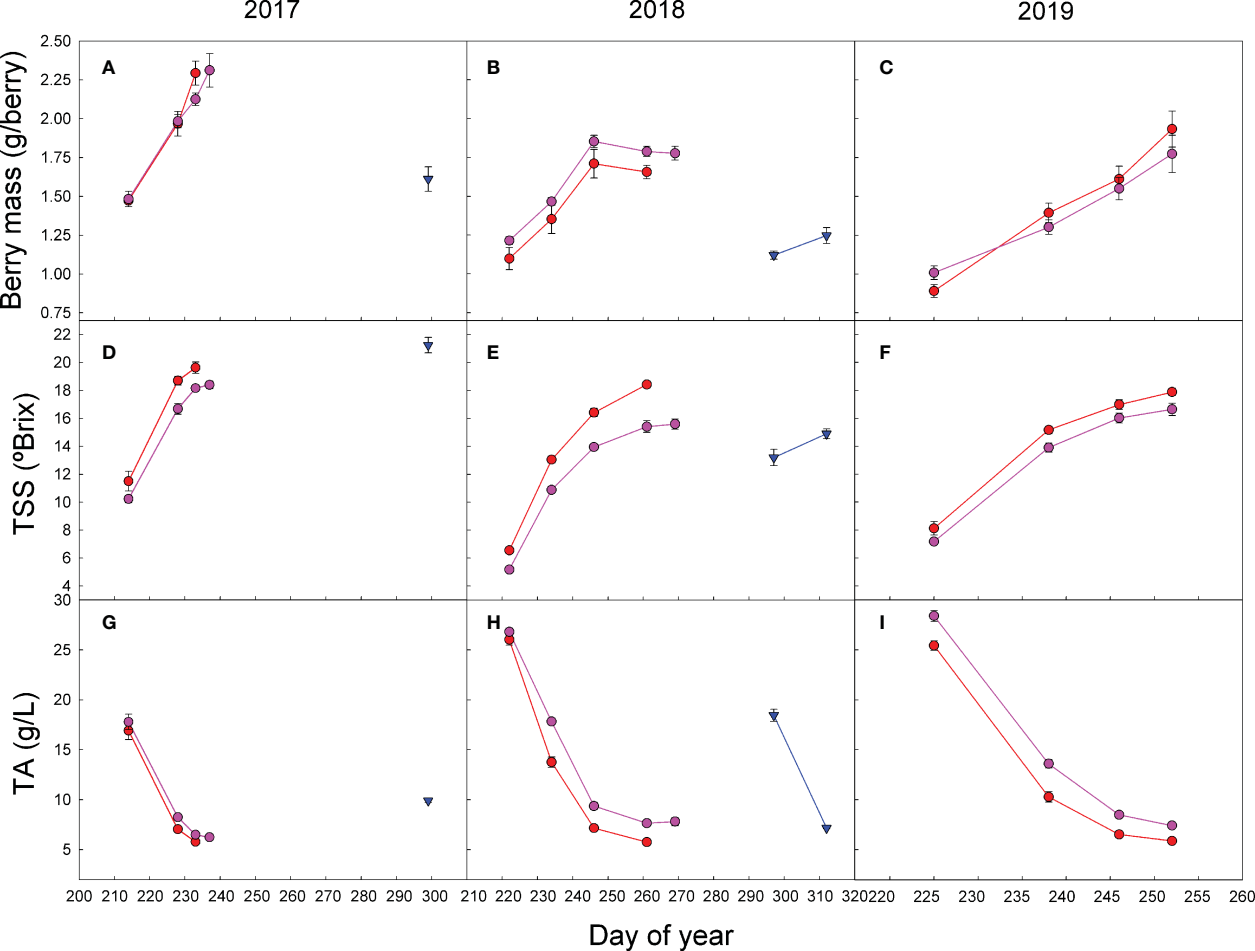
Effect of shading (•), double pruning (▼), and control (•) treatments on the seasonal evolution of **(A–C)** fresh berry mass; **(D–F)** total soluble solids (TSSs); and **(G–I)** the total acidity (TA) of ‘Macabeo’ grapes in **(A, D, G)** 2017, **(B, E, H)** 2018, and **(C, F, I)** 2019 seasons. Data are the average and SE of four values per treatment and date.

Differences in the TSS content at harvest were not consistent among treatments and seasons (**Table 2**, **Figure 4**). Despite the harvest criteria being the same for all treatments, aiming a level of 18°–19° Brix in the must, in 2017, the TSSs were significantly higher in the double pruning treatment than in the other two. However, in 2018, the TSS content at harvest was higher in the control than in the other treatments, despite the latter being harvested later in the season (**Table 2**, **Figure 4**). Regardless the level of grape ripeness reached by each treatment at harvest, the grape ripening kinetics showed that the control fruits had higher TSSs than the other treatments at each measurement dates (**Figure 4**). On the contrary, the level of TA during the ripening process tended to be lower in the control than in other treatments for the same date. In the 2017 harvest, TA was significantly higher in the double pruning treatment than in the control and shading ones (**Table 2**). In 2018 and 2019, however, the control treatment showed significantly lower TA. Must pH showed quite similar differences among treatments as the ones observed in TA, although not fully consistently (**Table 2**). This can be partially explained by the differences found in the tartaric-to-malic acid ratios between treatments. On the one hand, grapes from the control treatment tended to show lower levels of both tartaric and malic acid at harvest (**Table 2**), and, on the other hand, double pruning produced grapes with the highest malic acid levels. Thus, the tartaric-to-malic acid ratios were significantly lower in the double pruning treatment, followed by the shading one, and with the highest ratios found in the control. Similar effects were also found in the TSS-to-TA ratios (**Table 2**).

**Figure 5:**
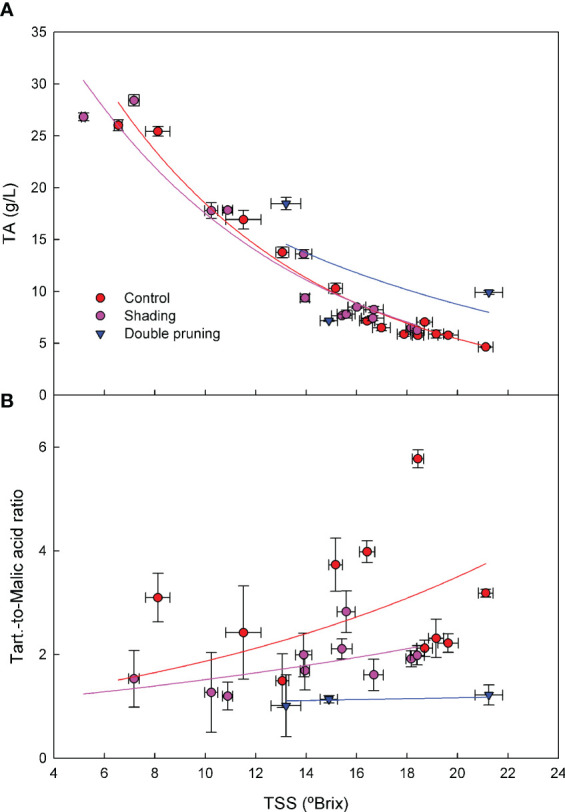
Effect of shading (•), double pruning, (▼) and control (•) treatments on the relationship between **(a)** TSSs and TA and **(b)** TSSs and tartaric-to-malic acid ratio for ‘Macabeo’ grapes in the 2017–2019 seasons. Data are the average of four replicates per treatment and date. TA, titratable acidity; TSSs, total soluble solids.

Regardless of the level of ripening at harvest, the relationship between TSSs and TA across the grape- ripening period in the 2017–2019 seasons was similar in the control and shading treatments (**Figure 5**). Double pruning, however, did show an overall effect of an increased TA for similar TSS levels than the control and shading treatments. In the tartaric-to-malic acid ratio, there was a clear effect of the treatments across seasons (**Figure 5**). This ratio was higher for the control than for the shading and for the shading than for the double pruning treatment.

Regarding wine composition, the differences among treatments in alcohol content (**Table 3**) were similar to those found in grape TSSs (**Table 2**). In any case, the TA and pH of the wines were significantly higher in the shading treatment as compared to the control, but the wines from the shading treatment had lower a TA than the double pruning one. Thus, the alcohol-to-acidity ratio was lower in the wines made from shaded and double-pruned vines as compared to that of the control. Similar effects as TA were found in 2017 in volatile acidity (VA), without significant differences between control and shading wines in 2018 and 2019 (**Table 3**). The citric acid content in wines was significantly higher in the wines from the double pruning treatment than in the ones from the control, which, in turn, had higher values than the ones from the shading treatment in every season. The malic acid content tended to be lower in the control than in the other treatments, while the opposite was observed in the lactic acid content (**Table 3**). Finally, the glycerol content was higher in wines from double-pruned vines than from that from the control or shading treatments. In 2017 and 2018, the glycerol content was significantly higher in control wines than in the shading treatment ones, whereas the opposite was observed in 2019 wines.

**Table 3:** Average values of wine composition at harvest over the 3 years of the experiment in ‘Macabeo’ grapevines subjected to different treatments.

Parameter	Year	Treatments											
		Control				Shading				Double pruning			
Alcohol (%)	2017	10.5	±	0.2	b	9.6	±	0.2	a	13.2	±	0.2	c
	2018	10.7	±	0.1	b	8.5	±	0.1	a	–		–	–
	2019	10.8	±	0.2	b	8.7	±	0.2	a	–		–	–
TA	2017	6.48	±	0.10	a	6.88	±	0.10	b	8.69	±	0.10	c
	2018	6.68	±	0.06	a	8.60	±	0.09	b	–		–	–
	2019	6.46	±	0.09	a	8.12	±	0.09	b	–		–	–
VA	2017	0.38	±	0.01	a	0.44	±	0.01	b	0.59	±	0.01	c
	2018	0.31	±	0.01	a	0.29	±	0.01	a	–		–	–
	2019	0.31	±	0.01	a	0.29	±	0.01	a	–		–	–
pH	2017	3.08	±	0.02	b	2.98	±	0.02	a	3.09	±	0.02	b
	2018	2.93	±	0.02	b	2.64	±	0.02	a	–		–	–
	2019	2.99	±	0.03	b	2.68	±	0.03	a	–		–	–
Citric acid (g/L)	2017	0.31	±	0.01	b	0.27	±	0.01	a	0.41	±	0.01	c
	2018	0.32	±	0.01	b	0.25	±	0.01	a	–		–	–
	2019	0.37	±	0.01	b	0.32	±	0.01	a	–		–	–
Malic acid (g/L)	2017	1.52	±	0.10	a	1.70	±	0.10	a	2.58	±	0.10	b
	2018	1.11	±	0.04	a	2.03	±	0.06	b	–		–	–
	2019	1.32	±	0.05	a	2.06	±	0.05	b	–		–	–
Lactic acid (g/L)	2017	0.61	±	0.02	b	0.59	±	0.02	b	0.48	±	0.02	a
	2018	0.65	±	0.02	b	0.45	±	0.03	a	–		–	–
	2019	0.66	±	0.02	b	0.40	±	0.02	a	–		–	–
Glycerol	2017	6.7	±	0.1	b	6.2	±	0.1	a	8.4	±	0.1	c
	2018	5.5	±	0.0	b	5.0	±	0.1	a	–		–	–
	2019	3.8	±	0.1	a	4.1	±	0.1	b	–		–	–
Alcohol-to-TA ratio (%/g/L)	2017	1.60	±	0.05	b	1.40	±	0.03	a	1.53	±	0.04	ab
	2018	1.68	±	0.04	b	1.10	±	0.03	a	–		–	–
	2019	1.62	±	0.02	b	1.00	±	0.03	a	–		–	–

Data are average and standard errors of four replicates per treatment and season (n = 4). Different letters mean a significant difference among treatments within each season (Duncan test; p < 0.05).

## 4 Discussion

In many Mediterranean-like climate conditions, the main agronomic and oenological challenges faced by wine producers are the low acidity and high pH of the grape must, rather than yield losses (Sadras et al., 2017). The present results have shown that through the use of field strategies, it is possible to modify the composition of grapes and significantly influence the quality of the base wines for the subsequent production of sparkling wines. Nevertheless, our results also showed an interactive effect between these practices and the season. In this sense, the effectiveness of these strategies was revealed since they allowed the microclimate of the cluster to be modified during the ripening phase, and the TSS-to-TA ratio to be reduced in grapes, and thus, the alcohol-acidity ratio in base wines for sparkling wine productions. Moreover, changes were observed in the content of different acids in the grapes and wines. Overall, the effect of the adaptation strategies evaluated was different in both the vine performance and grape ripening. Therefore, the effectiveness of these practices, as well as the combined use of shading + mulching, is individually discussed below, focusing on the effects of each strategy on the vine water status, vine performance, and grape and wine composition.

### 4.1 Shading effects

Grapevine shading reinforced the general idea that maximizing light interception particularly under high solar radiation conditions might not always optimize crop productivity (Corelli-Grappadelli and Lakso, 2007). Despite leaf or whole canopy gas exchange not determined in our experiment, vine water status was clearly improved by shading as compared to control, which perhaps helped to counteract the expected effect of lower light availability on the leaf photosynthesis rate and transpiration (Prieto et al., 2012; Prieto et al., 2020). Indeed, the photosynthetic response to light decreases under progressive water stress (Escalona et al., 2003). A field trial in which source-to-sink manipulation and irrigation strategies were investigated revealed that the vine water status was a more determinant factor influencing vine performance (Mirás-Avalos et al., 2017). In addition, a meta-analysis confirmed those results, highlighting that the effect of the water status was even clearer for white wines than for red ones (Mirás-Avalos and Intrigliolo, 2017). In our research, shading improved the vine water status by 0.2 MPa on average, as previously reported in other cultivars (Martínez-Lüscher et al., 2020). This effect did not allow vines to reach mildly severe water stress as recorded in control vines in the 2018 and 2019 experimental seasons. Indeed, the Ψ_stem_ values reached in the control treatment during the grape- ripening stage would indicate that water stress provoked severe damages to leaf function, thus impairing net photosynthesis up to thresholds at which the grapes would not ripen properly (Romero et al., 2010). In this regard, a recent study using photovoltaic panels that shaded 70% of the total incident solar radiation reported that the vine water status was also improved by 0.15–0.2 MPa, even if the vines were fully watered and able to maintain near-optimum water status conditions (Ferrara et al., 2023).

In our trial, in the last season after three consecutive years of 50% photosynthetically active radiation (PAR) exclusion, the shading treatment did not affect yield or pruning mass and, consequently, the Ravaz index, in comparison with the control vines. Only in 2018 did the rainy and cool conditions favor the development of fungal infections, which became more severe in the shading treatment. This is probably why there was a reduction in yield in the shading treatment as compared with the control in 2018. Nonetheless, it is worth noting that in this season, the control reached 24.8 tons/ha, which is more than double the amount allowed by the Cava Appellation of Origin for sparkling wine production. Therefore, the overall unaffected vine performance in response to such reduction in PAR suggests that improved light capture counterbalanced the irradiance reduction (González et al., 2019). Shoot fruitfulness was not affected by shading as compared to the control, which was intended by the installation of the nets after the formation of inflorescences (Lavee and May, 1997).

On the other hand, as intended, clear effects of shading on grape composition were recorded. The changes in microclimatic conditions in the shaded vines as compared to that of the control, were effective in delaying grape ripening and, consequently, harvest date. Not only the changes in the microclimate of the canopy and the cluster but also an additional ripening period (i.e., 1 week), under less hot weather conditions, allowed for a reduction of the TSS-to-TA ratio. This is in agreement with previous findings in a similar shading treatment applied to Cabernet-Sauvignon (Lu et al., 2021), in Pinot noir and Chardonnay (Ghiglieno et al., 2020), and in Riesling (Friedel et al., 2016). Nevertheless, this ratio was dependent on the ripeness level at harvest, which showed differences in TSSs among treatments. Thus, the relationship between TSSs and TA during ripening was not remarkably modified by the shading treatment as compared to the control. This suggests that although the shading treatment most likely reduced berry respiration and thus acid catabolism (Lu et al., 2021), it also reduced the grape’s accumulation of photoassimilates by photosynthesis to a similar extent (Hernández-Montes et al., 2022). In contrast, the relationship between tartaric and malic acids was significantly affected by shading. This was to be expected as a higher temperature and light intensity in the cluster zone generally results in an increase in the metabolic activity of the berries (Spayd et al., 2002; Sweetman et al., 2014). Nevertheless, the kinetics of malic acid, tartaric acid, pH, and potassium accumulation in grapes are not fully understood. A recent study points to potassium as a possible candidate to clarify the relationship between these parameters (Poni et al., 2018). Furthermore, the effect of temperature on tartaric acid and potassium, which, together with malic acid, determines the pH of must and wine remains to be explored.

The shading strategy, in our study, was effective in reducing wine pH as compared to that of the control in every season. The malic and lactic acid results are not fully conclusive as spontaneous malolactic fermentations seem to have taken place in some of the wines. However, the higher concentration of citric acid in the wines from the shading treatment confirms its effects of changes in acid synthesis and degradation in the grape as compared to the control.

### 4.2 Double pruning

Forcing vine regrowth during summer drastically shifted vine phenology and delayed grape ripening as compared to the other treatments. In addition, yield was reduced both in the season of application and in the subsequent one. Double pruning strongly affected vegetative growth and correct bunch formation. In fact, differences in yield components suggest that the main yield component affected by the double pruning technique was the number of berries per bunch. Nevertheless, the fruitfulness also decreased as compared to that of the control. This is in agreement with previous experiments that assessed this technique (Martínez de Toda et al., 2019; Martínez-Moreno et al., 2019; Cabral et al., 2023), and it may be attributable to a possible reduction in the nutritional reserves of the vines (Lebon et al., 2008; Smith and Holzapfel, 2009). Moreover, it should be noted that, in 2018, the double pruning vines suffered from powdery mildew during flowering, which can affect the fruit set percentage. Thus, the large shift in phenology provoked by this technique might increase the risk of suffering from fungal diseases. Moreover, given the large delay in the phenology caused by double pruning, the periods of maximum evapotranspirative demand occurred in different phenological stages than in the control or shaded vines. This may increase water requirements in forced vines as compared to the control, as Oliver-Manera et al. (2023) recently observed. Furthermore, they suggested that double-pruned vines were extremely sensitive to even mild water stress.

Regarding grape composition, our results clearly indicated that the TSS-to-TA ratio can be improved and, consequently, the alcohol-to-TA ratio. The later grape ripening confirmed that the temperature and humidity conditions in September and October were more favorable for a balanced grape composition. Thus, double pruning did increase the TA in relation to TSSs, and the tartaric-to-malic acid ratio in relation to TSSs, as other authors reported in other cultivars (Gu et al., 2012; Martínez de Toda et al., 2019; Martínez-Moreno et al., 2019; Oliver-Manera et al., 2023). These increases can be beneficial for wine stability as higher titratable acidity and a low tartaric-to-malic acid ratio decrease must pH. Glycerol content was also higher in the wines made from double-pruned grapes, which might be related to the enhancement of the smoothness sensation of the wine as it increases its density and viscosity (Silvestroni et al., 2018).

### 4.3 Combination of field strategies for climate change adaptation

Our research demonstrated the possibility of combining field strategies to add their effects. The Shading, together with the application of mulch, further reduced the water stress integral (S_Ψ_) from veraison to harvest during the 2018 and 2019 seasons as compared with Shading alone. Thus, both techniques, combined, had an additive effect in improving the vine water status. Shading likely reduced the evapotranspirative demand of the vines, while mulching reduced soil evaporation, as previously established (López-Urrea et al., 2020). This is particularly relevant today as the common agriculture policies economically support the application of organic mulching in vineyards in order to protect them from soil erosion and to improve the vineyard water balance, as demonstrated in the present study. Organic mulching could also be applied on the entire vineyard floor without a soil tillage operation (Buesa et al., 2021b). Moreover, other field strategies such as the application of sprinkler cooling within the cluster zone during ripening can also help reduce the impact of high temperatures (Caravia et al., 2017) beyond the improved water status of the techniques tested here, which may help to face heat waves. It is worth noting the positive effect that these techniques may have on soil water balance as the reduction in vine water stress is due to the reduction in evapotranspirative demand (Ramírez-Cuesta et al., 2023). Moreover, mulching could also affect root respiration and vine water status, due to not only soil water increases but also soil temperature modification (Hernández-Montes et al., 2017; Costa et al., 2018). Recently, Hernández-Montes et al. (2022) indeed highlighted the importance of respiratory processes on the carbon balance during vine phenology. For all of the above reasons, field adaptation strategies can be employed to face the detrimental effects of climate change in wine grapes. The two alternative practices tested differed both in terms of the infrastructure required, and the expected changes in vine phenology and development, and grape ripening. The high cost of installation of Shading nets may, however, preclude their final utilization, although the potential of this technique to minimize the risks of hail and wind damage and the impact of heat waves is also worth noting (Basile et al., 2014; Manja and Aoun, 2019; Martínez-Lüscher et al., 2020). The main limitation for the Double pruning strategy is the yield reduction and its carry-over effects, as it has been reported in other previous trials conducted with other wine grape varieties (Martínez de Toda et al., 2019; Cabral et al., 2023). This makes this technique profitable only for premium wine production of very high commercial value. However, the carry-over effects of this technique (Martínez-Moreno et al., 2019), together with its sensitivity to water deficit (Oliver-Manera et al., 2023), question its feasibility. On the other hand, the addition of mulching seems to be an easy and cheap technique to implement at the plot level, and with a proven potential to alleviate the impact of vine water stress (Buesa et al., 2021b). Moreover, the evaluated field strategies and their combination can be applied in the short-term. Nevertheless, the genetic material as a potential strategy should also be considered as a long-term adaptation (Medrano et al., 2015; Tortosa et al., 2016; van Leeuwen and Destrac-Irvine, 2017; Buesa et al., 2021c).

## 5 Conclusion

In the short term, grapevine adaptation to climate change can be achieved by using field strategies such as shading of the vines, double pruning, or shading + mulching, which can be all used to improve the composition of the wine base for making cava. The effectiveness of shading will depend to a large extent on the climatic conditions during the grape-ripening period, and more attention should be paid to the sanitary status of the grapes. In rainy and cool years, the application of the shading technique would not be recommended as it would be much more effective in hotter and drier vintages. On the other hand, double pruning can only be recommended for the production of premium Cava with a high commercial value, due to its drastic yield reduction and carry-over effects. All the investigated techniques are effective in improving the vine water status, and, in addition, the effect of the combination of shading and mulching was additive. This suggests the convenience of applying field practices in combination in light of future and more critical climate change scenarios and highlights the importance of further studies on the use of combined field adaptation strategies.

## Data availability statement

The raw data supporting the conclusions of this article will be made available by the authors, without undue reservation.

## Author contributions

IB and DI contributed to the conception and design of the study. IB, AY, DG, and FS acquired the data. IB, AY, and DI performed the data analysis and interpretation. IB prepared the first-draft. IB and DI reviewed and edited the manuscript. IB and DI supervised the work. IB and DI acquired the funding. All authors contributed to the article and approved the submitted version.

## Supplementary material

Supplementary Figure 1 Photographs of (A) the Shading and Control treatments and (B) the Double pruning treatment at the time of the second pruning.
