# Supplementary figures and images for: Testing field adaptation strategies for delaying grape ripening and improving wine composition in a cv. Macabeo Mediterranean vineyard
Source: Front Plant Sci. 2023 Apr 25;14:1155888. doi: 10.3389/fpls.2023.1155888 (PMC10167022; doi:10.3389/fpls.2023.1155888)

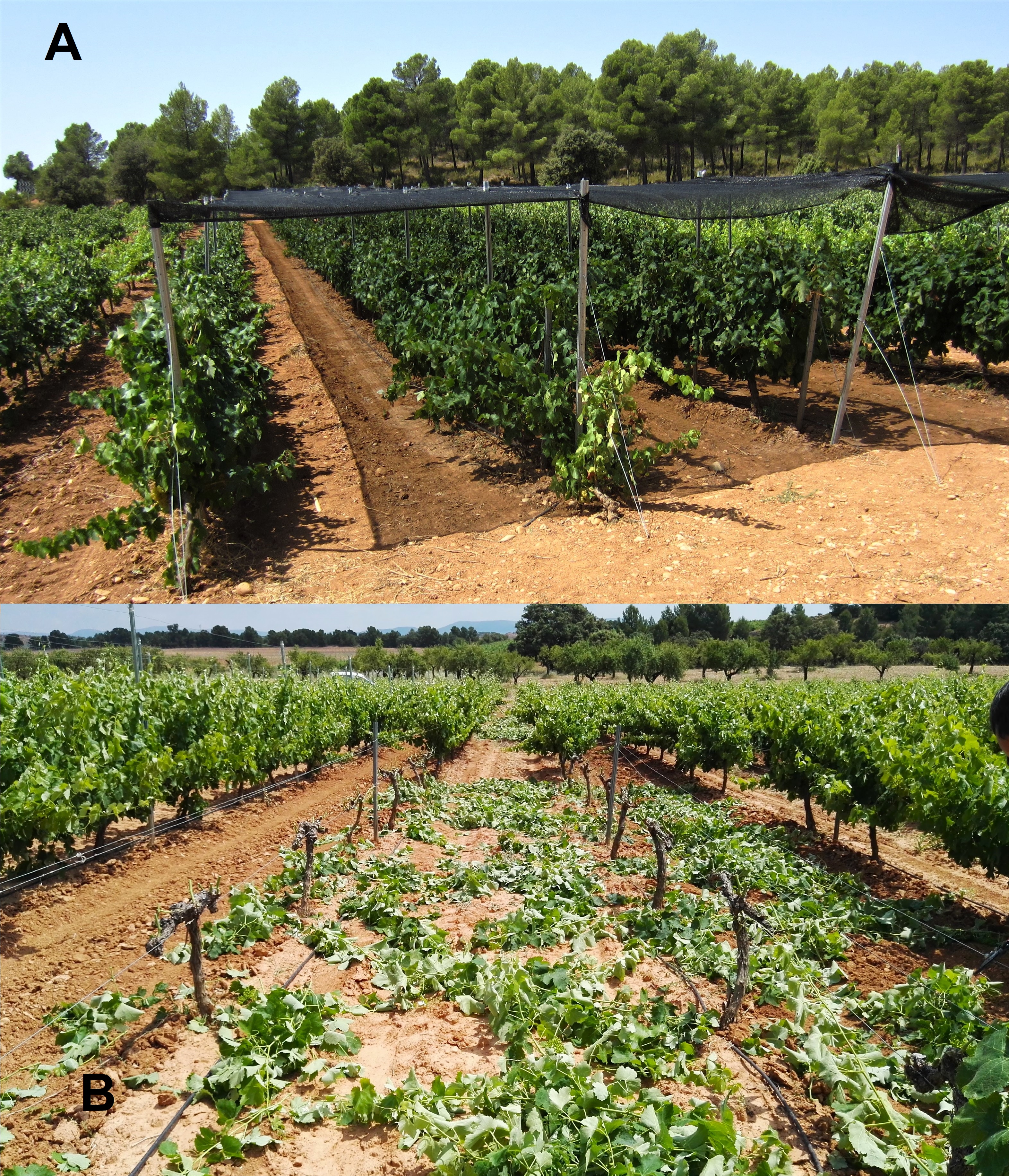

Supplement: Supplementary Figure 1 — Photographs of (A) the Shading and Control treatments and (B) the Double pruning treatment at the time of the second pruning. [file Image_1.jpeg]
